# Supplementary material for: Creation and annihilation of topological meron pairs in in-plane magnetized films
Source: Nat Commun. 2019 Dec 6;10:5603. doi: 10.1038/s41467-019-13642-z (PMC6898613; doi:10.1038/s41467-019-13642-z)
Supplement: Supplementary file 1 — Supplementary Information [file 41467_2019_13642_MOESM1_ESM.pdf]

Supplementary Information

Creation and annihilation of topological meron pairs in in-plane magnetized films

Gao et al.

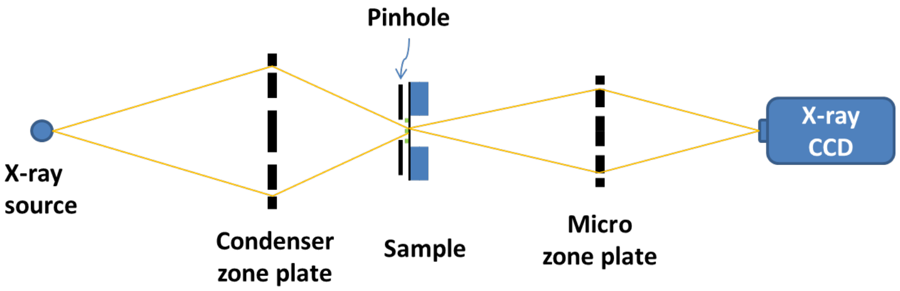

Supplementary Figure 1. Schematic drawing of MTXM setup.

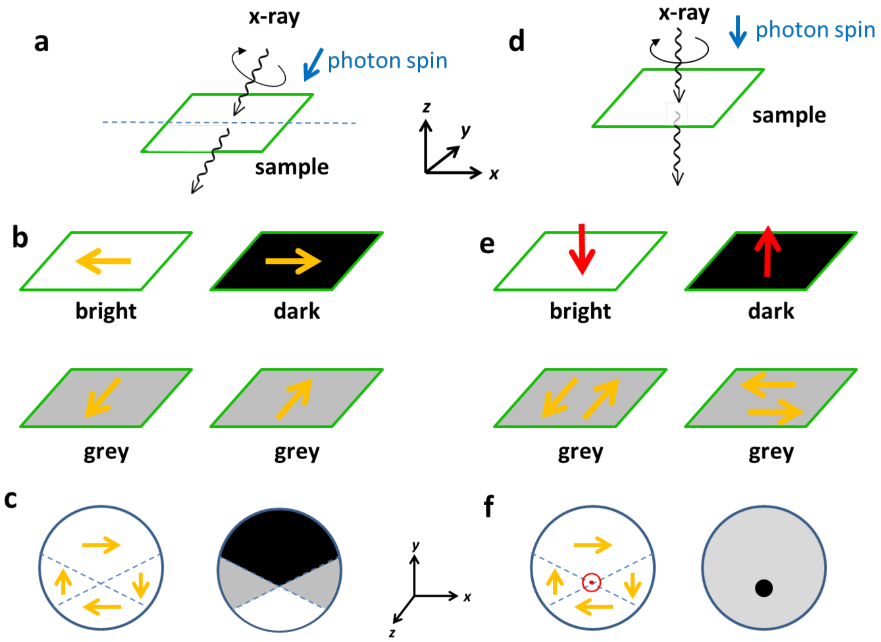

Supplementary Figure 2. Magnetic contrast in MTXM. **a~c**, By mounting the sample with a 30° sample tilting, different in-plane magnetization directions in a magnetic domain absorb a circular polarized x-ray beam differently due to the XMCD effect, leading to different magnetic contrasts (dark/bright/grey). **a,** the incident direction of x-ray. **b,** different contrasts (dark/bright/grey) from different magnetization directions (represented by the yellow arrows). **c**, An example of in-plane magnetic contrast of a vortex state in an FM disk. Note here only 4 directions (+*x*, -*x*, +*y*, -*y*) of magnetization and 3 magnetic contrasts (dark, grey, bright) are shown for simplicity. In real cases, however, both the magnetization and the corresponding contrast vary continuously as shown in Fig. 2 of the main text. **d~f**, By mounting the sample normal to incident x-ray, only the out-of-plane magnetization can give bright or dark contrast, and all the in-plane domains will show grey contrast. **d,** the incident direction of x-ray. **e,** the different contrast (represented by colors in the plane) of domains with different magnetization direction (represented by the yellow and red arrows for the in-plane and out-of-plane magnetizations, respectively). **f**, An example of out-of-plane magnetic contrast of a vortex state in an FM disk.

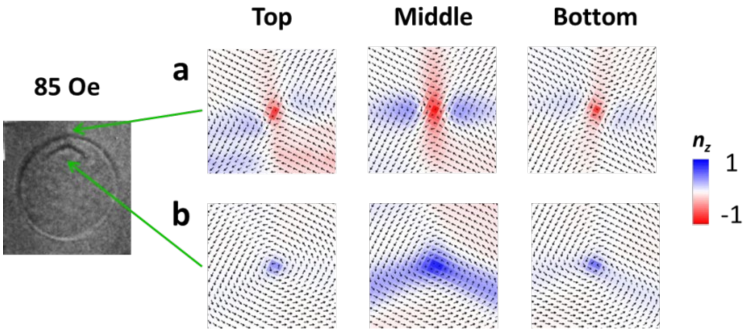

Supplementary Figure 3. Depth profile of the simulated spin textures in the sample at the top surface, middle plane, and bottom surface of the Py film, corresponding to the 85 Oe image of Fig. 4j in the main text. **a**, Magnetization profile around the antivortex core. **b**, magnetization profile around the vortex core. The in-plane magnetization profile is plotted by arrows and the out-of-plane component of the magnetization is represented by the colors.

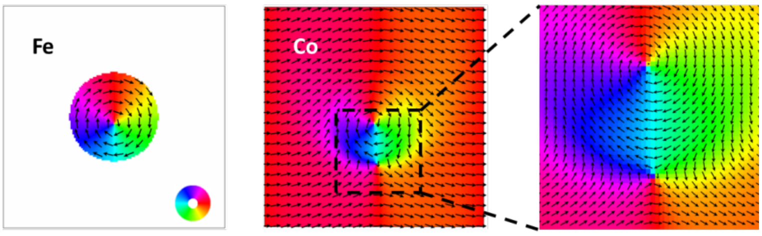

Supplementary Figure 4. A meron pair constructed in Co film by vortex imprinting from Fe disk.

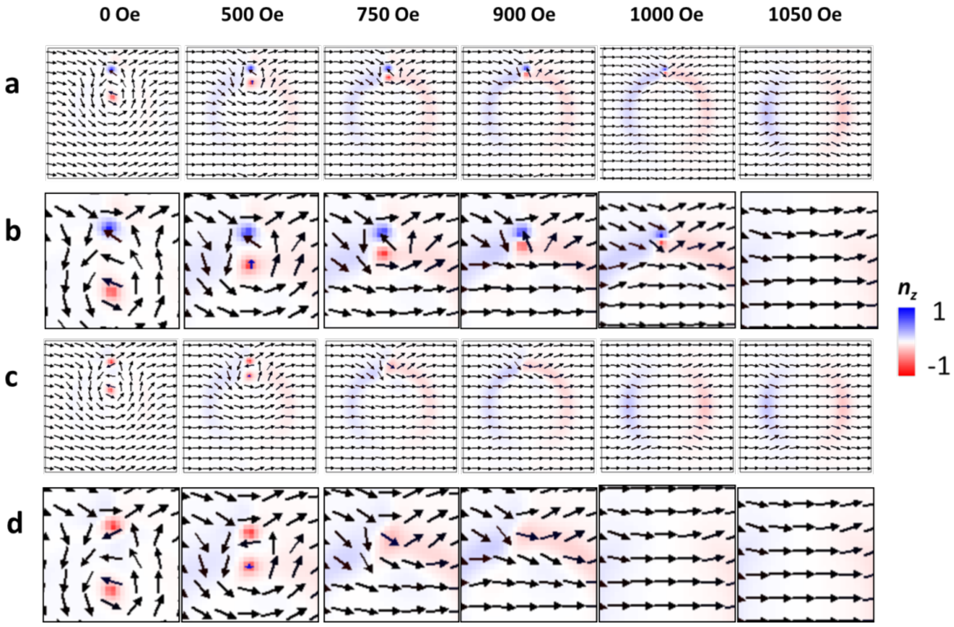

Supplementary Figure 5. Annihilation process of the meron pairs of different topologies in the Py film. **a,b**, the case of bimeron (*N*=-1). **c,d**, the case of meron-antimeron pair (*N*=0). The in-plane magnetization profile is shown by arrows with the color representing the out-of-plane component of the magnetization. Images in **b**(**d**) are the zoomed in profiles of the corresponding images around the meron cores in **a**(**c**).

The size of the simulation area is 600nm×600nm and the mesh size is $2.34\text{nm} \times 2.34\text{nm} \times 2.5\text{nm}$.

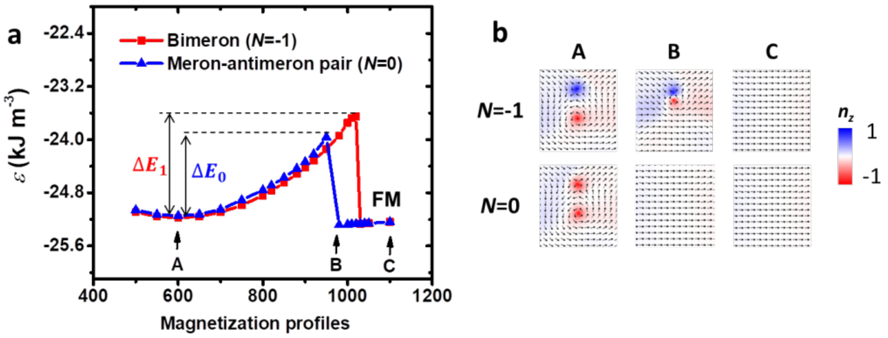

Supplementary Figure 6. a, The energy densities of the bimeron (N=-1) and the meron-antimeron pair (N=0) along the evolution paths to the final FM state (quasi single domain state) with the Zeeman energy calculated at H=600 Oe. The higher barrier for the bimeron (ΔE_1) than the meron-antimeron pair (ΔE_0) is a manifest of topological effect in the transition. The horizontal axis labels the magnetization profiles at the corresponding fields in Supplemenntary Figure 5 along the evolution path (e.g., the 500 magnetization profile corresponds to the magnetization profiles at H=500 Oe in Supplementary Figure 5). In addition, **b** gives representative zoomed in Py magnetization profiles at the three labeled locations (A,B,C) in the transition path.

Supplementary Note 1: Magnetic contrast of MTXM images

The setup of the full-field MTXM is illustrated in Supplementary Figure 1 [1]. The x-ray coming from the synchrotron source is focused by a zone plate (an x-ray version of optical lens) onto the sample placed closely after a pinhole for illumination. After transmitting through the sample, the x-ray is focused again by another zone plate onto the CCD camera. The setup is essentially an x-ray version of conventional optical microscope, with the visible light source replaced by the synchrotron x-rays, the optical condenser lens replaced by the x-ray condenser zone plate, and the optical objective lens replaced by the x-ray micro zone plate accordingly. Together with the XMCD effect, the MTXM achieves element-resolved magnetic imaging which has been widely used for the imaging of magnetic domains in magnetic nanostructures.

The XMCD effect is equivalent to the Magneto-Optic effect except the XMCD measurement is performed using x-rays with the x-ray photon energy equal to the L edge absorption energy of 3d transition metals so that the absorption is element specific. Akin to the Magneto-Optic Effect, left and right circular polarized x-rays will be absorbed differently by a FM material whose magnetization is parallel to the x-ray propagation direction. Equivalently, for a fixed circularly polarized x-rays, the ferromagnetic material will absorb the x-rays differently, depending on the projection the sample magnetization to the x-ray photon spin direction, so that magnetic domains with magnetization at different angles with respect to the x-ray photon spin can exhibit different contrast [2].

As shown in Supplementary Figures 2a and 2b, under the experimental condition of 30° sample tilting (i.e., 60° x-ray incident angle), the domains with magnetization pointing toward $+x$ and $-x$ directions will have dark and bright contrast, respectively, with magnetization pointing to $\pm y$ directions having grey contrast. Then a vortex state in an FM disk will exhibit a gradually changing contrast due to its curling magnetization (Supplementary Figure 2c). For normal incidence of x-rays (Supplementary Figures 2d and 2e), out-of-plane magnetizations in $+z$ and $-z$ directions show dark and bright contrast, while all the in-plane magnetizations give a grey contrast because they are all perpendicular to the x-ray photon spin. Therefore as schematically shown in Supplementary Figure 2f, normal incidence of x-rays is best to image the vortex core polarities.

Supplementary Note 2: Deformation of vortex and antivortex cores

Domain walls in FM thin films of tens of nanometer thickness have the tendency of titling the wall magnetization towards the out-of-plane direction, leading to the so-called asymmetric Bloch and Néel walls [3]. The asymmetric Bloch walls have been recently confirmed in Py disks [4]. The Py thin film in this work has 80nm thickness thus should experience a similar tendency of tilting the magnetization towards the out-of-plane direction, leading to elongated tails in the out-of-plane magnetic contrast around the vortex or antivortex cores in Figs. 4j~4l of the main text. Using micromagnetic simulation, we investigated in details of this titling effect.

Supplementary Figure 3 shows the profiles of the simulation results around the vortex and antivortex cores at the top surface, middle plane, and bottom surface of the Py film in our sample. Both the vortex and antivortex structures are well maintained throughout the whole film thickness with little change of the core positions. The in-plane magnetization texture around the cores, however, has a noticeable depth dependence which results in an out-of-plane titling of the magnetization to form an asymmetric Néel wall [3]. This out-of-plane component is the origin of the tail around the cores observed in Fig. 4j of the main text. In fact, the existence of the tail helps the identification of the core polarity. From Supplementary Figure 3, it is easy to see that the topological number and in-plane winding number of the spin texture are well preserved despite the deformation. In particular, the topology number as define by Eq (1) in the main text is robust against any continuous deformations.

Supplementary Note 3: Universality of the meron pair stabilization

Because the stabilization of the meron pair in an in-plane magnetized film is guaranteed by the winding number conservation, our method does not depend too much on the material choices, as long as vortex imprinting occurs. As an example, Supplementary Figure 4 shows a simulation of a 10nm Co film with a Fe disk of 40nm thickness and 100nm radius on top. Similar to Fig. 1c of the main text, this system can also support a stable meron pair. We further varied the materials of the disk/film (Py/Co, Ni/Fe, Co/Fe, and Co/Ni, etc.) and found that all of them support the stable meron pairs as far as a vortex can be stabilized in the disk [5, 6] and imprinted into the film.

Supplementary Note 4: Enhanced topological effect in the annihilation process with a different sample geometry

Noticing the universality of the skyrmion construction discussed above, we further explored possible enhancement of the topological effect by reducing the Co disk radius and the Py thickness. We performed a simulation for a system with Co disk arrays (40nm thickness, 200nm radius, 600nm center-to-center distance) on a 40nm thickness Py film. Supplementary Figure 5 shows the simulated meron pair annihilation process at the bottom Py surface from both an N=-1 and an N=0 initial states at different magnetic field. The result shows clearly a stronger field for the annihilation of the N=-1 bimeron than the field for the annihilation of the N=0 trivial pair. The difference is ~300 Oe, much greater than the ~10 Oe difference for the sample shown in Fig. 5 of the main text. We don't have a specific reason for this enhancement, and future theoretical study is definitely needed.

Supplementary Note 5: Discussion about the additional energy barrier associated with topological transitions

Transition between two topological states is an active and difficult research topic. In particular, a transition from an initial topological magnetic state (N=1 or N=0) to a FM state (quasi single domain state, N=0) usually needs to overcome an energy barrier which separates the two local energy minimum (or called valley in energy landscape). As shown by many recent theoretical works [7, 8, 9], the difficulty on this subject is that there exist infinite paths (e.g., magnetization profile sequences) to connect these two local states, making it impossible to find the exact minimal energy path linking the topological state to the FM state. Different methods have been developed aiming to approach the minimal energy path in order to estimate the energy barrier [10, 11, 12]. One practical approach is to take the series of magnetization profiles from the actual annihilation process as the transition path and calculate their energies at fixed system parameters to obtain the corresponding energy barrier. For our case, this corresponds to the calculation of the energy densities of the whole sequence of magnetization profiles for the annihilation processes of the bimeron state (N=-1) and the meron-antimeron pair states (N=0) for example shown in Supplementary Figure 5, but with the Zeeman energy calculated using a prefixed magnetic field (because this field is a system parameter upon which the energy landscape depends on), and obtain the corresponding energy barrier which separates the bimeron state (or the meron-antimeron pair) and the quasi single domain state (FM state).

Since our goal is to compare the difference between the bimeron and the meron-antimeron pair, we calculate the energy densities of the series of the magnetization profiles shown in Supplementary Figure 5 (plus many detailed intermediate steps) with the Zeeman energy calculated at the field of H=600 Oe which is below the annihilation field of the meron pairs to best present the energy barrier that separates the two energy valleys in the configuration space. Supplementary Figure 6 depicts the calculated energy density for bimeron (E_1, red squares) and meron-antimeron pair (E_0, blue triangles) along the evolution path of the magnetization profiles (Supplementary Figure 5). Each evolution shows that the initial state needs to overcome an energy barrier ($\Delta E_0 = 1.18\,\mathrm{kJ\,m^{-3}}$ for meron-antimeron pair, and $\Delta E_1 = 1.53\,\mathrm{kJ\,m^{-3}}$ for bimeron) to collapse into the final FM state. The role played by topology can be seen by comparing the energy barriers between these two paths, which shows that the bimeron path has a higher energy barrier than the meron-antimeron pair path by $\Delta E_1 - \Delta E_0 = 0.35\,\mathrm{kJ\,m^{-3}}$. One can interpret this additional energy barrier as the manifest of topological effect since it will take a higher magnetic field to trigger the topological transition (N=-1 to N=0) compared with the non-topological transition (N=0 to N=0). We note that such manifestation of topology is in accordance with the result of previous works [13, 14, 15] which also show the different annihilations of N=1 and N=0 states into the final FM state.

Supplementary References

1. Fischer, P. et al. Soft X-ray microscopy of nanomagnetism. *Mater. Today* **9**, 26-33 (2006).
2. https://www-ssrl.slac.stanford.edu/stohr/xmcd.htm
3. Hubert, A. & Schäfer, R. Magnetic domains: the analysis of magnetic microstructures (Springer, Berlin, 1998).
4. Im, M. Y. et al. Dynamics of the Bloch point in an asymmetric permalloy disk. *Nat. Commun.* **10**, 593 (2019).
5. Schneider, M., Hoffmann, H., Otto, S., Haug T., & Zweck, J. Stability of magnetic vortices in flat submicron permalloy cylinders. *J. Appl. Phys.* **92**, 1466-1472 (2002).
6. Guslienko K Y et al. Magnetization reversal due to vortex nucleation, displacement, and annihilation in submicron ferromagnetic dot arrays. *Phys. Rev. B* **65**, 024414 (2001).
7. Hagemeister, J. Stability of single skyrmionic bits. *Nat. Commun.* **6**, 8455 (2015).
8. Rohart S., Miltat J., & Thiaville A. Path to collapse for an isolated Néel skyrmion. *Phys. Rev. B* **93**, 214412 (2016).
9. Cortés-Ortuño, D. et al. Thermal stability and topological protection of skyrmions in nanotracks. *Sci. Rep.* **7**, 4060 (2017).
10. Henkelman, G., Uberuaga, B. P., & Jónsson, H. A climbing image nudged elastic band method for finding saddle points and minimum energy paths. *J. Chem. Phys.* **113**, 9978-9985 (2000).
11. Heistracher, P., Abert, C., Bruckner, F., Vogler, C., & Suess, D. GPU-accelerated atomistic energy barrier calculations of skyrmion annihilations. *IEEE Trans. Magn.* **54**, 7206105 (2018).
12. Müller, Gideon P., et al. Duplication, Collapse, and Escape of Magnetic Skyrmions Revealed Using a Systematic Saddle Point Search Method. *Phys. Rev. Lett.* **121**, 197202 (2018).
13. Hertel, R., & Schneider, C. M. Exchange explosions: Magnetization dynamics during vortex-antivortex annihilation. *Phys. Rev. Lett.* **97**, 177202 (2006).
14. Li, J. et al. Tailoring the topology of an artificial magnetic skyrmion. *Nat. Commun*. **5**, 4704 (2014)..
15. Stier, M., Häusler, W., Posske, T., Gurski, G., & Thorwart, M. Skyrmion–anti-skyrmion pair creation by in-plane currents. *Phys. Rev. Lett.* **118**, 267203 (2017).
